# The role of HLA antigens in recurrent primary focal segmental glomerulosclerosis

**DOI:** 10.3389/fimmu.2023.1124249

**Published:** 2023-02-23

**Authors:** Ibrahim Batal, Pascale Khairallah, Astrid Weins, Nicole K. Andeen, Michael B. Stokes

**Affiliations:** ^1^ Pathology and Cell Biology, Columbia University Irving Medical Center, New York, NY, United States; ^2^ Medicine, Division of Nephrology, Columbia University Irving Medical Center, New York, NY, United States; ^3^ Pathology, Brigham and Women’s Hospital and Harvard Medical School, Boston, MA, United States; ^4^ Pathology, Oregon Health & Science University, Portland, OR, United States

**Keywords:** recurrent focal segmental glomerular sclerosis, kidney allograft, HLA, diffuse podocytopathy, focal segment glomerulosclerosis

## Abstract

Primary focal segmental glomerulosclerosis (FSGS), typically characterized by diffuse podocyte foot process effacement and nephrotic syndrome (diffuse podocytopathy), is generally attributed to a circulating permeability factor. Primary FSGS can recur after transplantation where it manifests as diffuse foot process effacement in the early stages, with subsequent evolution of segmental sclerotic lesions. Previous published literature has been limited by the lack of stringent selection criteria to define primary FSGS. Although immunogenetic factors play an important role in many glomerular diseases, their role in recurrent primary FSGS post-transplantation has not been systematically investigated. To address this, we retrospectively studied a multicenter cohort of 74 kidney allograft recipients with end stage kidney disease due to primary FSGS, confirmed by clinical and histologic parameters. After adjusting for race/ethnicity, there was a numeric higher frequency of HLA-A30 antigen in primary FSGS (19%) compared to each of 22,490 healthy controls (7%, adjusted OR=2.0, P=0.04) and 296 deceased kidney donors (10%, OR=2.1, P=0.03). Within the group of transplant patients with end stage kidney disease due to primary FSGS, donor HLA-A30 was associated with recurrent disease (OR=9.1, P=0.02). Multivariable time-to-event analyses revealed that recipients who self-identified as Black people had lower risk of recurrent disease, probably reflecting enrichment of these recipients with *APOL1* high-risk genotypes. These findings suggest a role for recipient and donor immunogenetic makeup in recurrent primary FSGS post-transplantation. Further larger studies in well-defined cohorts of primary FSGS that include high-resolution HLA typing and genome-wide association are necessary to refine these hereditary signals.

## Introduction

Focal segmental glomerulosclerosis (FSGS) is a histologic pattern of glomerular scarring associated with diverse forms of kidney damage (1). Primary FSGS is characterized by diffuse foot process effacement with nephrotic syndrome (diffuse podocytopathy; DP). The pathogenesis of primary FSGS remain unknown but clinical and experimental evidence support the existence of circulating permeability factor(s) that causes primary diffuse podocyte injury (1), which, lead to podocyte loss, segmental adhesion of the injured tuft to Bowman’s capsule (segmental glomerulosclerosis) and eventually to global glomerulosclerosis (2). Since these putative permeability factors remain poorly characterized, the diagnosis of primary FSGS currently relies on clinical and pathologic findings, and exclusion of known secondary causes.

Primary FSGS can recur following kidney transplantation and has a negative impact on graft survival. Recurrent primary FSGS manifests initially as nephrotic syndrome and diffuse foot process effacement, identical to minimal change disease (MCD), while segmental sclerotic lesions develop later (3, 4), probably reflecting cumulative podocyte depletion in susceptible individuals (5).

In addition to primary FSGS, the 2021 Kidney Disease Improving Global Outcomes (KDIGO) classification of FSGS includes three other categories (1) Secondary FSGS, caused by viruses, drugs, or, more commonly, adaptive responses to reduction of functioning nephrons and compensatory glomerular hyperfiltration, usually associated with focal foot process effacement and subnephrotic proteinuria (2) Genetic FSGS, which is most commonly monogenic in nature, and include familial, syndromic, and sporadic cases, and (3) Undetermined FSGS, characterized by focal foot process effacement, proteinuria without the nephrotic syndrome, and no identified secondary or genetic causes (6).

However, the current classification of FSGS is not ideal. The diagnosis of secondary FSGS can be difficult as, depending on the time of presentation, viral and drug-induced causes may go unnoticed. Diagnosing genetic FSGS is also challenging. While the most common monogenic causes of FSGS can now be efficiently identified using targeted gene panels, the list of genetic causes is continually expanding (7). Furthermore, it is increasingly apparent that common inherited variants, such as HLA polymorphisms, may also contribute to the risk of primary FSGS (8), which complicates the current definition of genetic FSGS. Equally important, classifying FSGS in patients with Apolipoprotein L1 (*APOL1*) kidney risk variants is particularly problematic (9). Nephropathic *APOL1* variants occur with high frequency but low penetrance in populations with recent African ancestry, and may contribute to a higher incidence of kidney diseases from diverse etiologies (e.g., HIV-associated nephropathy, COVID-19 associated nephropathy, lupus nephritis, and hypertensive nephrosclerosis) (10, 11). That said, investigators from Mayo Clinic have shown that defining primary FSGS based on the presence of nephrotic syndrome and >80% foot process effacement is effective in excluding adaptive and monogenic forms of FSGS (12).

Despite the negative impact of recurrent DP on allograft survival (13), our knowledge of the immunologic and inherited factors associated with disease recurrence is extremely limited. This reflects the small sample size of published studies, the difficulty of completely excluding non-primary forms of FSGS, and the fact that FSGS in the allograft may arise from etiologies other than recurrent disease (e.g., adaptive FSGS from reduced nephron number and drug toxicity). In this report, we use the more expansive term DP to include cases of early recurrent primary FSGS that are characterized by nephrotic syndrome with complete or near-complete foot process effacement (≥ 80% of glomerular capillary surface area) but lack segmental sclerosis on light microscopy (14, 15).

Previous studies by us and others showed an association between donor inherited factors and recurrence of both membranous nephropathy (16, 17) and IgA nephropathy (18). We hypothesized that immunogenetic background in the donors and/or the recipients contributes to the development of recurrent DP/primary FSGS. In this report, we examined the frequency of HLA antigens in a large multicenter cohort of kidney transplant patients with end stage kidney disease (ESKD) attributed to DP/primary FSGS. Here for the first time we show that patients with ESKD due to primary FSGS tend to be enriched with HLA-A30 antigen while donor HLA-A30 is associated with increased risk of recurrent disease after transplantation.

## Material and methods

This study was a retrospective multicenter study that included the pathology archives of three North American medical centers: Columbia University Irving Medical Center (CUIMC, New York, NY), Brigham and Women’s Hospital (BWH, Boston, MA), and Oregon Health & Science University (OHSU, Portland, OR). Each center collected data under approval by their Institutional Review Boards.

In this report, strict inclusion criteria were used to select our cohort:

(1) Recurrent DP (n=47) was defined as recurrence of nephrotic syndrome (proteinuria ≥ 3.5 g and serum albumin ≤ 3.5 g/dL) in kidney allograft recipients with ESKD attributed to DP/FSGS, accompanied by allograft biopsies (performed in all but one recipient) showing foot process effacement involving ≥ 80% of glomerular capillary surface area. The remaining patient had a biopsy-proven MCD in the native kidney followed by FSGS, and presented 8 days after transplantation with nephrotic syndrome (urine protein/creatinine: 10 g/g and serum albumin 1.6 g/dL) and serum creatinine of 1.1 mg/dL. Native kidney biopsies and/or pathology reports were available for 29 of these subjects and showed FSGS (n=21) and MCD (n=8, including 7 patients with subsequent native kidney biopsies that showed FSGS). The remaining 18 cases had a clinical diagnosis of FSGS but pathologic materials were not available for re-review.

(2) Non-recurrent DP (n=27) was defined as absence of nephrotic syndrome post-transplantation in kidney transplant recipients who developed nephrotic syndrome in their native kidney (≥ 3.5 g of proteinuria and ≤ 3.5 g/dL of serum albumin) with a concurrent native kidney biopsy showing near complete foot process effacement (involving ≥ 80% of the glomerular capillary surface area). Of these, 24 subjects had native kidney biopsy showing FSGS and 3 demonstrated MCD (including 2 patients with subsequent native biopsies showing development of FSGS).

Serologic and/or molecular typing for HLA-A, -B, -DR, and -DQ was performed for both donors and recipients. The outcome was recurrence of DP in the kidney allograft and the subjects were censored at loss of follow-up or allograft failure. Studied variables including recipient demographics (age, sex, and race), donor demographics, allograft source, HLA-mismatch (A, B, and DR: scale 0-6), prior kidney transplant, and induction immunotherapy, were extracted from the medical record.

To test which HLA antigens that are potentially associated with DP, the most frequent HLA antigens in our transplant cohort were compared to two group of controls:

(1) External controls of healthy US residents (n=22,490) from National Bone Marrow Donor Program (19), providing HLA allelic frequencies for reference comparisons.

(2) Internal controls of deceased kidney donors whose kidneys were transplanted at CUIMC in 2010 onwards that were matched with our cohort of DP patients with regard to self-reported ancestry (n=296; for each patient with DP, four deceased donors were matched by ancestry).

Statistical analyses were performed using Prism 9 (Graphpad Inc, San Diego, CA) and SPSS Statistics, 26.0 (IBM, Armonk, NY). Categorical data were compared using Fisher Exact test while continuous data were compared using the Mann-Whitney test. With the except of discovery study, P less than 0.05 with two-sided hypothesis testing were considered statistically significant.

## Results

### Basic characteristics of transplant patients with ESKD attributed to DP/primary FSGS

We identified 74 kidney allograft recipients who had ESKD due to DP/primary FSGS [CUIMC (n=60), OHSU (n=9), and BWH (n=5)]. Subjects were followed-up for a median of 68 (IQRs: 17, 107) months after transplantation. During follow-up, 47 (64%) patients developed recurrent disease [median 34 (IQRs: 12, 210) days post-transplantation] and 40 (54%) developed allograft failure [median 26 (IQRs: 14, 70) months after transplantation].

The median age at transplantation was 35 years (**Supplemental Table 1**). The cohort included 51% women, 27% Black recipients, 9% with prior kidney transplant, 40% recipients of living-related allografts, and a median donor-recipient HLA mismatch of 4. Donors had a median age of 35 years and included 57% women and 27% Black subjects. Most of the patients received induction therapy with a depleting agent (83%; including 76% with Thymoglobulin and 7% with Alemtuzumab) (**Supplemental Table 1**).

### DP/Primary FSGS in the native kidney is associated with high frequency of HLA-A30 antigen

We initially identified the most frequent HLA antigens in all 74 recipients in this cohort [A2: 45%, A1: 20%, and A30: 19% in the A region; B44: 19%, B51: 18%, and B35: 16% in the B region; DR15: 28%, DR4: 27%, and DR7: 24% in the DR region; and DQ6: 47%, DQ7: 41%, and DQ2: 29% in the DQ region] (**Supplemental Table 2**).

**Figure 1:**
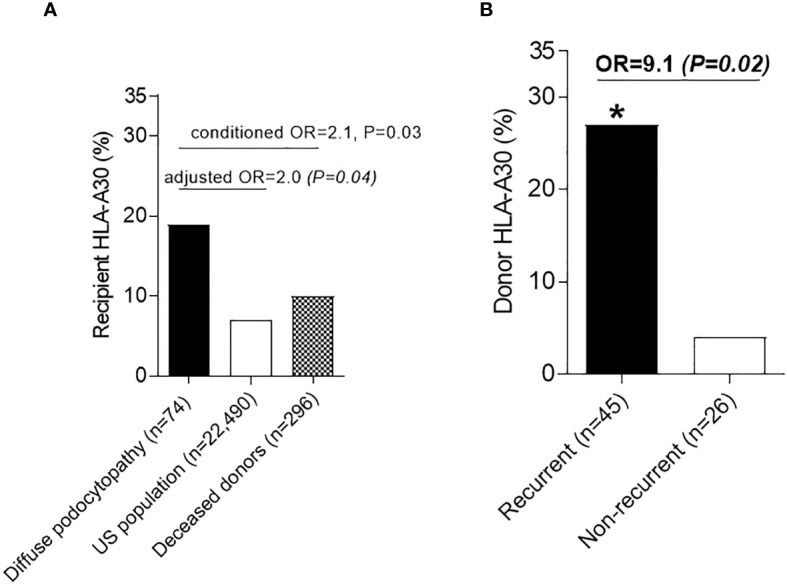
Human Leukocyte antigens in diffuse podocytopathy **(A)** Prevalence of HLA-A30 antigen in cases with diffuse podocytopathy (patients with end stage kidney disease due to diffuse podocytopathy, n=74) compared to US population (n=22,490) and deceased kidney donors matched to cases with regard to race/ethnicity (n=296). **(B)** Prevalence of HLA-A30 in the kidney donors of patients with recurrent (n=42) vs. non-recurrent (n=22) diffuse podocytopathy. Donor typing for class-I was not available for 2 donors with recurrent diseases and 1 without recurrent disease. * (significant P value).

We compared the frequency of these antigens to external healthy controls (n=22,490) (19). Since 12 HLA antigens have been compared, a Bonferroni-corrected significance cutoff of 0.05/12 = 0.004 was utilized to define statistical significance. HLA-A30 was the only antigen that was more prevalent in recipients with DP (OR=3.0, P=0.0008). However, this particular antigen, which is typically more prevalent in Black people (**Supplemental Table 2**), did not reach Bonferroni-threshold for statistical significance after adjusting for race/ethnicity using Cochran–Mantel–Haenszel test (adjusted OR=2.0, P=0.04) (**Figure 1A**). Using conditional logistic regression conditioned on self-reported race/ethnicity matching, HLA-A30 was more common in DP compared to a second set of controls composed of 296 deceased kidney donors matched by race/ethnicity [14/74 (19%) vs. 29/296 (10%), OR=2.1, P=0.03] (**Figure 1A**).

### HLA-A30 antigen in the donor is associated with recurrent DP/primary FSGS

To test whether HLA-A30 is associated with recurrent DP, we compared the frequencies of this antigen in both recipients and donors with recurrent and non-recurrent disease. While the proportion of recipient HLA-A30 was not significantly different [6/47 (13%) recurrent vs. 8/27 (30%) non-recurrent, P=0.12], the frequency of donor HLA-A30 was higher in recurrent disease [12/45 (27%) recurrent DP vs. 1/26 (4%) non-recurrent DP, OR=9.1, P=0.02)] (**Figure 1B**). Despite the small sample size, the results for the association between HLA-A30 and recurrent disease remained significant even after adjustment for donor age and donor Black race (binary logistic regression: OR=11.0, P=0.03). The frequency of other HLA antigens did not differ between recurrent and non-recurrent diseases (**Supplemental Table 3**). Overall, donor HLA-A30 had 96% specificity, 27% sensitivity, 92% positive predictive value, and 43% negative predictive value for predicting recurrence of DP (**Supplemental Table 4**). The breakdown for the association of HLA-A30 in the recipients and donors with recurrent disease is also presented in **Supplemental Table 5**.

### Black recipients have lower risk of developing recurrent DP/primary FSGS

To identify independent predictors for recurrent DP, all transplant patients with ESKD due to DP (n=74) were studied in a time-to event model, where DP recurrence was considered as the outcome of interest (**Table 1**). Univariable and multivariable analyses showed that Black recipient race was the only independent variable associated with the outcome, where it was protective against recurrent disease [(aHR)=0.31, P=0.008] (**Table 1**). When the relation between recipient Black recipients and recurrent DP was explored further, we found that 4/5 (80%) of self-identified Black recipients with non-recurrent disease that were genotyped for *APOL1* had high-risk genotypes (3 G1/G1 and 1 G1/G2).

**Table 1:** Univariable and multivariable analyses of the associations with recurrent disease in patients with end stage kidney disease due to diffuse podocytopathy.

Variables	Univariable (n=74)			Multivariable (n=71), events=45	
	N events	HR (95% CI)	P value	aHR (95% CI)	P value
Recipient age at transplant (per each year)	47	0.99 (0.97 – 1.01)	0.49		
Recipient female gender	47	1.21 (0.68 – 2.16)	0.51		
Black recipient	47	0.26 (0.11 – 0.62)	0.002	0.31 (0.13 – 0.74)	0.008
Black donor	45	0.68 (0.34 – 1.38)	0.28		
Allograft source (living-related)	47	1.44 (0.81 – 2.56)	0.21		
Number of HLA mismatches (per antigen: 0-6)	45	0.81 (0.64 – 1.01)	0.07	0.86 (0.67 – 1.10)	0.22
Recipient HLA-A30	47	0.52 (0.22 – 1.23)	0.14		
Donor HLA-A30	45	1.90 (0.97 – 3.71)	0.06	1.66 (0.85 – 3.24)	0.14
Prior kidney transplantation	47	1.06 (0.42 – 2.69)	0.90		
Induction therapy with depletion agent	36	0.55 (0.25 – 1.21)	0.14		

Cox proportional hazards models were constructed to account for confounders. All factors that demonstrated a suggestive association with the outcome (P value <0.1) at the univariable analysis were included in the multivariable cox proportional hazards models. Individuals with missing information on a tested predictor were excluded from the corresponding univariable time-to-event analysis; individuals with missing data in one or more predictors at the multivariable analyses were also excluded from the latter analyses.

## Discussion and perspectives

Primary FSGS and MCD are DP, characterized by diffuse foot process effacement and nephrotic syndrome (6). In the native kidney, there is growing recognition that abnormalities of T and B cell immunity are common in DP and may play a pathogenic role (20). This includes the association with allergy, immunization, and B cell neoplasms, as well as responsiveness to immunomodulatory therapies, including corticosteroid therapy and B cell depletion (21, 22). With regard to inherited factors, genome-wide association studies (GWAS) have shown that steroid-sensitive nephrotic syndrome, including MCD and primary FSGS, is associated with genomic susceptibility loci in HLA regions (23–25) and non-HLA regions (23, 26), most of which are linked to the immune system. Lastly, in addition to other associations, nephropathic *APOL1* genotypes have also been linked to FSGS (27), sometimes with diffuse foot process effacement. Together, these findings suggest that DP/primary FSGS is a polygenic disease associated with immune dysregulation and immunogenetic susceptibility.

Although the pathogenesis of recurrent DP post-transplantation is unknown, the existence of a circulating glomerular permeability factor is supported by several observations, including rapid recurrence of the nephrotic syndrome following transplantation, induction of proteinuria in the rat using serum from a patient with recurrent FSGS (28), and resolution of recurrent nephrotic syndrome following re-transplantation of the kidney into a different recipient (29).

Uffing et al. published the largest study to date of recurrent FSGS in the kidney allograft, which included 176 kidney transplant recipients who developed ESKD from FSGS (13). In addition to the association with White recipient race, older recipient age, and history of bilateral nephrectomy, this study showed a significant association between recurrent FSGS and lower recipient body mass index (BMI) (13). The latter finding may reflect the association between higher BMI and adaptive FSGS secondary to obesity in the native kidney, which is unlikely to recur early after transplantation in the form of nephrotic syndrome. Interestingly, a few prior studies have shown that young recipient age, rather than old recipient age as shown by Uffing et al., was a risk factor for FSGS recurrence (30, 31). Furthermore, depending on the study, recurrence rate for primary FSGS in the kidney allograft varies from 20% to 52% of cases (32, 33). These contradicting results suggest contamination of the previously studied cohorts by secondary forms of FSGS and support the crucial need for using rigorous criteria to exclude non-primary cases of FSGS when studying disease recurrence after transplantation.

Although HLA antigens have emerged as important immunogenetic risk factors in many immune-mediated kidney diseases (34–37), their role in recurrent disease has not been systematically examined in a pure cohort of DP/primary FSGS. One study attempted to assess this issue in a pediatric transplant population with ESKD attributed to FSGS and found an association between recurrent FSGS and a few HLA class-II antigens, including DQ2, DR7, and DR53 (38). However, that study was registry-based and lacked data on clinical and histologic parameters at time of diagnosis of FSGS in the native kidney, which further increases the possibility of selection bias and contamination by non-primary forms of FSGS, which in our experience comprise the bulk of cases labeled as FSGS-induced ESKD.

In this report, we used stringent inclusion criteria requiring the presence of both nephrotic syndrome (≥ 3.5 g of proteinuria, ≤ 3.5 g/dL serum albumin) and ≥ 80% of foot process effacement to assemble a relatively pure cohort of DP/primary FSGS (15). Despite the contribution of three major transplant centers, we only could identify 74 transplant patients fulfilling our rigorous selection criteria. Notably, our study showed that several patients with ESKD due to primary FSGS had prior native kidney biopsies that demonstrated MCD. These findings further support the intimate association between these two morphologic manifestations of DP.

Although not reaching statistical significance by Bonferroni criteria in this relatively small sample, our data suggested an association between primary FSGS in the native kidney and HLA-A30 antigen. Prior studies have shown an association between HLA-A30 and several autoimmune diseases, including type 1 diabetes mellitus, vitiligo, and systemic sclerosis (39–41), which suggest that HLA-A30 may contribute to immune dysregulation. Our study also demonstrated an association between HLA-A30 in the donor and recurrent primary FSGS. It is conceivable that donor HLA antigens may influence antigen presentation by donor immune cells or podocytes, which can function as antigen-presenting cells (42), cross reactivity/molecular mimicry, or acceleration of podocyte death in immunologically susceptible patients.

Despite the high positive predictive value (92%) of HLA-A30 in predicting recurrent disease, it was not surprising that this relatively low frequency HLA antigen, which has a prevalence of 7% in the general population and 19% in patients with ESKD secondary to primary FSGS, did not reach statistical significance as an independent predictor for recurrent disease in this small cohort.

With regard to recipient inherited factors, a few prior studies have shown that White recipient race is a risk factor for developing recurrent FSGS (13, 43, 44). We propose that White recipient race may not be a real risk factor but rather may be confounded by the genetic make-up in Black recipients, in whom FSGS may be less likely to recur after receiving a kidney from a donor with a different genomic constitution. Indeed, multivariable analyses in the current study showed lower risk of recurrence in Black subjects with FSGS/DP. This might reflect the increased prevalence of high-risk *APOL1* genotypes (80%) in Black recipients with non-recurrent DP. It is now well accepted that donor *APOL1* kidney risk variants may be a risk factor for podocyte damage in the kidney allograft (45, 46) while *APOL1* status in the recipient is less likely to influence recurrent disease. Importantly, while it is still unclear how to classify FSGS in patients with *APOL1* high-risk genotypes, it would be interesting in the future to systematically assess *APOL1* genotypes in recipients with vs. without recurrent DP/primary FSGS.

Our findings should be interpreted in light of several limitations, including small sample size, low resolution of HLA typing, lack of HLA-DQ typing in several recipients and donors, lack of *APOL1* genotyping in most Black recipients, and the lack of detailed clinical history (e.g., recipient BMI and whether native kidney nephrectomies were performed or not).

In conclusion, a strict definition of recurrent DP/primary FSGS is an initial step to advance our understanding of recurrent disease after transplantation. Our pilot study represents the first attempt to systematically assess immunogenetic predictors of recurrent disease in a well-defined cohort of transplant patients with ESKD attributed to DP/primary FSGS. Our results suggest that donor HLA-A30 is a potential risk factors for recurrent DP/primary FSGS while Black recipient race may be a potential protective factor. However, it is still possible that the observed association between HLA-A30 and primary FSGS is affected by the differential distribution of HLA-A30 across worldwide populations. Therefore, future larger GWAS studies that properly control for genetic ancestry are crucial to examine the effects of HLA-A30 in a more comprehensive manner, to refine the donor and recipient genomic signals contributing to recurrent primary FSGS, and to obtain better insights into the pathobiology of recurrent DP (47). The ultimate goals of such efforts would be to guide the clinician towards improving donor-recipient matching and develop targeted approaches to prevent recurrence and improve allograft survival.

## Data availability statement

The original contributions presented in the study are included in the article/**Supplementary Material**. Further inquiries can be directed to the corresponding author.

## Ethics statement

The studies involving human participants were reviewed and approved by Columbia University Institutional Review Board. Written informed consent from the participants’ legal guardian/next of kin was not required to participate in this study in accordance with the national legislation and the institutional requirements.

## Author contributions

IB participated in research design, performance of research, data analyses, and in the writing of the manuscript. PK participated in performance of research and writing of the manuscript. AW participated in research design, performance of research, and writing the manuscript. NA participated in research design, performance of research, and writing the manuscript. MBS participated in research design and writing of manuscript. PK is currently at the Baylor College of Medicine Houston, TX. All authors contributed to the article and approved the submitted version.
