# Supplementary material for: The role of HLA antigens in recurrent primary focal segmental glomerulosclerosis
Source: Front Immunol. 2023 Feb 23;14:1124249. doi: 10.3389/fimmu.2023.1124249 (PMC9995699; doi:10.3389/fimmu.2023.1124249)
Supplement: Supplementary file 1 [file Table_1.pdf]

Supplementary Material

Supplemental Table 1: Demographics and clinical characteristics for patients with end stage kidney disease due to diffuse podocytopathy

	**Total (n=74)**	**Recurrent DP (n=47)**	**Non-recurrent DP (n=27)**	**P values (recurrent vs. not)**
Age at transplant (yrs)	35 (23, 46)	35 (20, 46)	34 (26, 45)	0.79
< 30 yrs	26/74 (35%)	16/47 (34%)	10/27 (37%)	0.81
30-50 yrs	35/74 (47%)	22/47 (47%)	13/27 (48%)	1.00
>50 yrs	13/74 (18%)	9/47 (19%)	4/27 (15%)	0.76
Female sex	38/74 (51%)	26/47 (55%)	12/27 (44%)	0.47
Recipient race				
-White	42/74 (57%)	34/47 (72%)	8/27 (29%)	0.0006
-Black	20/74 (27%)	6/47 (13%)	14/27 (52%)	0.0008
-Hispanic/Latinx	11/74 (15%)	7/47 (15%)	4/27 (15%)	1.00
-Other	1/74 (1%)	0/47 (0%)	1/27 (4%)	0.37
Allograft Source				
-Living Related	30/74 (40%)	22/47 (47%)	8/27 (30%)	0.22
-Living Unrelated	16/74 (22%)	11/47 (23%)	5/27 (18%)	0.77
-Deceased Donor	28/74 (38%)	14/47 (30%)	14/27 (52%)	0.08
Donor age at transplant (yrs)[1]	35 (24, 46)	40 (29, 49)	29 (21, 41)	0.02
< 30 yrs	25/68 (37%)	12/43 (28%)	13/25 (52%)	0.07
30-50 yrs	30/68 (44%)	20/43 (47%)	10/25 (40%)	0.62
>50 yrs	13/68 (19%)	11/43 (25%)	2/25 (8%)	0.11
Donor female sex[2]	39/68 (57%)	28/43 (65%)	11/25 (44%)	0.13
Donor race [3]				
-White	42/71 (59%)	30/45 (67%)	12/26 (46%)	0.13
- Black	19/71 (27%)	10/45 (22%)	9/26 (35%)	0.28
- Hispanic/Latinx	9/71 (13%)	5/45 (11%)	4/26 (15%)	0.72
-Other	1/71 (1%)	0/45 (0%)	1/26 (4%)	0.37
#HLA mismatches (0-6) [4]	4 (3, 5)	3 (3, 4)	4 (3, 5)	0.06
Prior Kidney transplants	7/74 (9%)	5/47 (11%)	2/27 (7%)	1.00
Induction therapy [5]				
-Thymoglobulin	46/60 (76%)	25/36 (70%)	21/24 (88%)	0.13
-Alemtuzumab	4/60 (7%)	3/36 (8%)	1/24 (4%)	0.64
-IL25R inhibitors	6/60 (10%)	5/36 (14%)	1/24 (4%)	0.39
-No induction	4/60 (7%)	3/36 (8%)	1/24 (4%)	0.64

HLA mismatch is calculated based on A, B, and DR antigens

[1] Data on donor age was not available for 6 patients (4 recurrent and 2 non-recurrent)
[2] Data on donor sex was not available for 6 patients (4 recurrent and 2 non-recurrent)
[3] Information on donor race/ancestry was not available for 3 patients (2 recurrent and 1 non-recurrent)
[4] Information on HLA mismatch was not available for 3 patients (2 recurrent and 1 non-recurrent)
[5] Information on induction therapy was not available for 14 patients (11 recurrent and 3 non-recurrent)

Supplementary Table 2: Prevalence of HLA antigens in external controls and transplant recipients who had end stage kidney disease due to diffuse podocytopathy

HLA antigens	Recipients with diffuse podocytopathy (n=74)	US population controls (n= 22,490)	Unadjusted P values	Adjusted P values
HLA-A2	33/74 (45%)	10,950/ 22,490 (49%)	0.49	
HLA-A1	15/74 (20%)	5,711/ 22,490 (25%)	0.35	
HLA-A30	14/74 (19%)	1,635/ 22,490 (7%)	0.0008	0.04
HLA-B44	14/74 (19%)	5,138/ 22,490 (23%)	0.49	
HLA-B51	13/74 (18%)	2,149/ 22,490 (10%)	0.03	
HLA-B35	12/74 (16%)	3,926/ 22,490 (17%)	0.9	
HLA-DR15	21/74 (28%)	5,821/ 22,490 (26%)	0.60	
HLA-DR4	20/74 (27%)	6,288/ 22,490 (28%)	0.99	
HLA-DR7	18/74 (24%)	5,045/ 22,490 (22%)	0.68	
HLA-DQ6	28/59 (47%)*	9,705/ 22,490 (43%)	0.51	
HLA-DQ7	24/59 (41%)*	7,597/ 22,490 (34%)	0.27	
HLA-DQ2	17/59 (29%)*	8,540/ 22,490 (38%)	0.18	

US population controls including 15,740 (70%) White subjects, 2,308 (10%) Black subjects, 2,170 (10%) Hispanic/Latinx subjects, and 2,272 (10%) others. In this cohort, HLA-A30 was most prevalent in Black subjects (574, 25%) followed by Hispanic/Latinx (225; 10%), White (726; 5%), and others (110; 5%)

Since 12 HLA antigens were compared, Bonferroni-corrected cutoff of 0.004 was considered significant

* DQ typing was only available for 59 of kidney allograft recipients with end stage kidney disease due to diffuse podocytopathy

Supplementary Table 3: Prevalence of other donor HLA antigens in transplant patients with end stage kidney disease due to diffuse podocytopathy

HLA antigens	Total donors	Recurrent	Non-recurrent	P values (recurrent vs. non-recurrent)
HLA-A2	32/71 (45%)	22/45 (49%)	10/26 (39%)	0.46
HLA-A1	15/71 (21%)	9/45 (20%)	6/26 (23%)	0.77
HLA-B44	20/71 (28%)	11/45 (24%)	9/26 (35%)	0.42
HLA-B51	7/71 (10%)	4/45 (9%)	3/26 (12%)	0.7
HLA-B35	13/71 (18%)	6/45 (13%)	7/26 (27%)	0.21
HLA-DR15	14/71 (20%)	10/45 (22%)	4/26 (15%)	0.55
HLA-DR4	9/71 (13%)	5/45 (11%)	4/26 (15%)	0.72
HLA-DR7	23/71 (32%)	17/45 (38%)	6/26 (23%)	0.29
HLA-DQ6	22/54 (41%)	17/35 (49%)	5/19 (26%)	0.15
HLA-DQ7	20/54 (37%)	10/35 (29%)	10/19 (53%)	0.14
HLA-DQ2	25/54 (46%)	20/35 (57%)	5/19 (26%)	0.05

Supplementary Table 4: Sensitivity, specificity, positive predictive value, and negative predictive value of donor HLA-30 in predicting recurrent diffuse podocytopathy

	**Recurrent diffuse podocytopathy**	**Non-recurrent diffuse podocytopathy**
**Donors HLA-A30**	12	1
**Donors without HLA-A30**	33	25

* Among the total cohort of 74 kidney allograft subjects with end stage kidney disease native due to diffuse podocytopathy, HLA-A typing was available for 71 donors (45 recurrent and 26 non-recurrent disease)

Sensitivity: 12/45 = 27%

Specificity: 25/26 = 96%

Positive predictive value: 12/13 = 92%

Negative predictive value: 25/58 = 43%

Supplementary Table 5: The association of donor and recipient HLA-A30 with recurrent disease in subgroups of transplant patients with end stage kidney disease due to diffuse podocytopathy classified according to the presence of HLA-A30 in the recipients and the donors

**Subgroups**	**Recurrence rate**
Recipient HLA-A30$^-$/Donor HLA-A30$^+$ (n=9)	9/9 (100%)
Recipient HLA-A30$^+$/Donor HLA30$^+$ (n=4)	3/4 (75%)
Recipient HLA-A30$^-$/Donor HLA-A30$^-$ (n=48)	30/48 (63%)
Recipient HLA-A30$^+$/Donor HLA-A30$^-$ (n=10)	3/10 (30%)

HLA-A typing was available for 71 donor-recipient pairs (45 recurrent and 26 non-recurrent disease)
